# Women escaping domestic violence to achieve safe housing: an integrative review

**DOI:** 10.1186/s12905-024-03143-7

**Published:** 2024-05-31

**Authors:** Virginia Stulz, Lyn Francis, Anshu Naidu, Rebecca O’Reilly

**Affiliations:** 1 Faculty of Health, School of Nursing and Midwifery, University of Canberra, Building 10, Office 10B7, 11 Kirinari St, Bruce, Canberra, ACT 2617 Australia; 2 Western Sydney University, PO Box 63, Penrith, Sydney, NSW 2751 Australia; 3 Western Sydney University, School of Nursing and Midwifery, Sydney, NSW 2745 Australia; 4 Faculty of Health Sciences, Australian Catholic University, School of Nursing, Midwifery and Paramedicine, 40 Edward St, North Sydney, Sydney, NSW 2060 Australia

**Keywords:** Experiences, Integrative review, Intimate partner violence, Domestic violence, Safe housing

## Abstract

**Background:**

This integrative review summarises original research that explores women’s experiences of escaping domestic violence to achieve safe housing.

**Methods:**

Integrative review. A robust search strategy was conducted using the following databases: Scopus, Cumulative Index to Nursing and Allied Health (CINAHL), Cochrane, Medline and PubMed. All articles were assessed for quality using the Mixed Methods Appraisal Tools (MMAT) scoring. Whittemore and Knafl’s (2005) five stage approach was used to analyse the primary literature related to women’s and stakeholders’ experiences of escaping domestic violence to achieve safe housing.

**Results:**

A total of 41 articles were retrieved and 12 papers were included in this review (six qualitative, one quantitative and five mixed methods) that fulfilled the inclusion criteria. Four overarching themes were identified: ‘Experiences of leaving domestic violence’, ‘Barriers to achieving safe housing’, ‘Facilitators to achieving safe housing’ and ‘The road to recovery’. The ‘Experiences of leaving domestic violence’ theme included two subthemes: ‘the losses’ and ‘ongoing contact with the perpetrator’. The ‘Barriers to achieving safe housing’ theme included three subthemes: ‘financial insecurity’, ‘being judged by others for leaving and service availability’. The ‘Facilitators to achieving safe housing’ theme included two sub-themes: ‘support, partnership, and collaboration between women and service providers’ and ‘feeling respected and heard’. The ‘Road to recovery’ theme included two sub-themes: ‘being a good mother’ and ‘empowerment after leaving domestic violence’.

**Conclusions:**

This review has highlighted the need for service and health care providers to work together and collaborate effectively with the woman experiencing and escaping domestic violence, especially in rural and remote areas. This means giving women access to the most suitable educational resources and services that are appropriate for their unique situation. Tailoring support for women is crucial to enable women to achieve safe housing and to be able to live a safe life with their children, away from the perpetrator of the domestic violence.

## Background

Violence perpetrated towards women by current or previous intimate partners often leads to dislocation, homelessness, isolation and lack of support for the woman and, if a mother, her children. Domestic violence (DV) is violence that occurs between current or previous intimate partners in the form of physical and/or sexual violence, emotional abuse, or coercive control [1]. The term DV is used interchangeably with other terms such as intimate partner violence (IPV), abuse against women, domestic and family violence (D&FV). In relation to including ‘family violence’, this extends the context of the violence to between all family members, and not purely intimate partners [1]. To align with the aim of this paper, the term DV and/or IPV will be used throughout, with the exception of direct quotes.

Women and children are disproportionately affected by male-perpetrated violence [2, 3]. Despite having government programs such as Staying Home Leaving Violence Program and national organisations to address domestic violence [4–6], government reports recognise that supporting women within their homes is not always possible. As DV impacts on women’s housing stability [7], rehoming women and children is a priority however, when rehoming women and their children, community connections and social support are crucial to consider.

A Domestic Violence Crises Service (DVCS) report, Staying Home after Domestic Violence, found that more than 37% (of 35 women whose cases were analysed) were unable to sustain long-term residency in their family homes following the end of the violent relationship. Over 50% of the women who were homeowners or private renters had lost their homes within 12 months of the separation [8]. Furthermore, due to the lack of affordable and safe accommodation many women and children remain in violent environments or resort to insecure and potentially unsafe accommodation to escape the violence [8].

DV is a primary contributor to illness, disability, and death for Australian women between the ages of 18–44 [9]. One woman is murdered by her current or former partner every week in Australia and this risk of extreme violence and homicide is higher for Indigenous Australian women [10]. Being in a relationship with a violent partner has detrimental impact on financial security [7, 11], and mental health [12]. Moreover, DV reframes how women understand themselves and their identity negatively, decreasing their self-esteem and sense of agency [13].

Experiencing DV complicates a mother’s role and identity as a mother which intensifies the effects of violence on their lives and that of their children [14]. The perpetrator’s coercive behaviours can threaten the mother’s wellbeing and undermine her parenting ability and the relationship shared with her children [15]. There is no guarantee that leaving a violent partner will stop the violence [16]. In fact, for many women and children, it exacerbates the risk of harm [11]. The challenges of leaving a violent partner are compounded for mothers who also have to help children transition into a new life and deal with trauma [17]. Other than children, safety of their pets is another factor that prevents DV victims from leaving their homes to seek their own safety [18]. As pets are often seen as family members and survivors often view their pets as a form of support, separation is made more difficult [18]. Pets are often involved in DV situations and need to be considered in resources, programs and safety plans for women experiencing DV [19].

Health care practitioners have identified that they value woman-centred care when working with women who have experienced DV and these attributes included asking questions directly, responding holistically and supporting the woman’s choice. Health care practitioners have also identified that midwives are the most appropriate health providers to conduct screening for women experiencing DV and social workers are most suitable for providing a comprehensive response. They have identified support needs as working with a team, knowing their role when working with women experiencing DV and training and mentoring programs [19]. Adults and children experiencing DV have been able to access the “Orange Door” in Brimbank Melton in Victoria. The ‘Orange Door” is a partnership between non-government organisations, Aboriginal community services, Western Health and the Labour Government [20]. Additionally, the Family Violence Multi-Agency Risk Assessment Framework (MARAM) [21] in Victoria ensures services are effectively identifying, assessing and managing family violence risk. The aim of MARAM is to increase the wellbeing and safety of Victorians by ensuring services can effectively identify and manage DV risk [21]. The MARAM framework has also been evaluated recently and there is solid evidence that it has been broadly effective [22].

Transient accommodation may thus be required with a multi-service, wraparound approach that supports the woman and her children to seek alternate, safe and permanent housing, and promote recovery of holistic well-being. Collaboration between specialised DV services, police, child protection, social services, health professionals, mental health care, legal services, culturally specific services, and housing services is necessary in responding to the immediate crisis as well as providing follow up care in the post crisis stage [8]. Such services must work together to provide holistic, individualised and tailored support and service provision for each woman and child experiencing DV. There is much evidence to support that keeping women and children within their established community, and involving the community itself to provide a wraparound, multi-pronged approach to service delivery has multiple benefits. These include improving women’s and children’s social support and belongingness that ultimately result in improved mental health and reduced psychological distress; as well as increasing community awareness about DV and how to best support a known victim-survivor [23–26].

Economic abuse by perpetrators has been linked to economic hardship and women who have experienced high levels of emotional and physical abuse have also experienced increased economic hardship. It is important to support survivors to identify strategies for maintaining social supports and developing programs to provide tangible resources to decrease women’s material hardship experiences [27]. Financial control inducing financial insecurity is a form of domestic violence and causes more uncertainty about leaving a DV situation.

### Aim of the integrative review

The aim of the integrative review was to explore women’s experiences of escaping DV and achieving safe housing.

#### Objectives

1. To describe women’s experiences of escaping DV and achieving safe housing.
2. To explore barriers and facilitators to escaping DV and achieving safe housing, from the perspectives of women.

## Methods

### Design

This study adopted a comprehensive literature search strategy and analysis of articles which met the inclusion criteria using the approach advocated by Whittemore and Knafl [28]. The six stages of this integrative review approach enabled a rigorous and comprehensive review incorporating the following: problem identification; literature search; data evaluation; data analysis and presentation of the studies’ characteristics and writing the final integrative review. Using the Whittemore and Knafl [28] approach we identified primary research articles which included six qualitative and four mixed methods studies.

### Problem identification

DV reframes negatively how women understand themselves and their identity, decreasing their self-esteem and sense of agency [13]. Furthermore, experiencing DV, complicates a mother’s role and identity as a mother, which intensifies the effects of violence on their lives and that of their children [14]. Health care practitioners should be aware of how they can support women experiencing DV to safer housing and demonstrate ‘readiness’ in their roles to assist women in these situations. Understanding the support systems and processes of how women leave DV and IPV situations will contribute to this ‘readiness’ of health care practitioners working with women.

### Literature search

Online databases searched included Cumulative Index of Nursing and Allied Health (CINAHL), Cochrane, Medline, Pubmed and Scopus. Articles included peer-reviewed quantitative, qualitative or mixed methods journal articles that were published from 2011 to April 2024 in the English language. The Population Intervention Comparison Outcome (PICO) framework was used to determine correct search parameters. Prior to finalising the review, we conducted another search in November 2023, to identify additional papers published in 2022–2023, but there were no papers found. The population of interest included women, the intervention of interest was safe housing, there was no comparison group and the outcomes of interest included women’s experiences of escaping DV and achieving safe housing. Table 1 provides detail of the inclusion and exclusion criteria. An example of the search terms are shown in Table 2.

**Table 1:** Inclusion / exclusion criteria

Inclusion criteria	Exclusion criteria
Published between 2011 to 2024	Published prior to 2011
Published in English language	Published in language other than English
Primary research article	Articles other than primary research
Qualitative, quantitative & mixed method articles	Systematic reviews, protocols / guidelines, literature reviews
Related to women’s experiences of escaping DV	Articles not focussing on achieving safe housing for women experiencing DV

**Table 2:** Search terms used

Domestic violence OR DV OR "partner violence" OR "family violence" OR "battered women" OR "spouse abuse” OR "intimate partner violence" AND
experience*AND
Wom?n AND
Safe house* OR safe hous?ng OR "safe houses" OR "safe housing"

### Search outcome

**Fig. 1:**
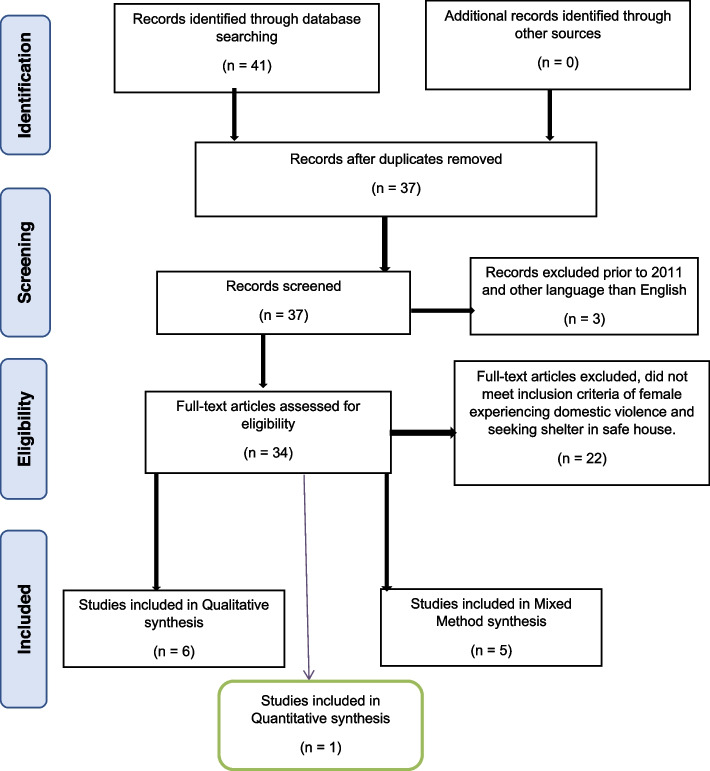
Search strategy using PRISMA flow diagram

Initial search results generated 41 records identified across all databases. We searched for the abstract and the title. After four duplicates were removed using the Endnote referencing system and manual checking list, 37 articles remained. A total of three articles were removed as they were published prior to 2011 and not in English, leaving 34 articles that were assessed for eligibility. Twenty-two articles were excluded because they did not meet the inclusion criteria of women experiencing DV and seeking shelter in a safe house. In total, 12 articles (six qualitative, one quantitative and five mixed methods) remained in the final review. All included articles focused on the experiences of women leaving DV or IPV situations and seeking safe housing. This robust literature search strategy was conducted using the Preferred Reporting Items for Systematic reviews and Meta-Analysis (PRISMA) flow diagram (see Fig. 1).

### Data extraction and evaluation

Data from the 12 articles were extracted including: aim of the study, country, design and methods, sample, data analysis, findings, and the impact of women’s experience in accessing shelter after experiencing DV. All authors evaluated the articles using the Mixed Methods Appraisal Tools (MMAT) [29] for the six qualitative, one quantitative, and five mixed methods studies (see Table 3).

**Table 3:** Characteristics of the qualitative and mixed method studies

Qualitative studies							
**First author Year & Country**	**Title and Aim of the study**	**Participants in study**	**Study Design**	**Data collection**	**Data analysis**	**Findings / outcomes (if reported)**	**Quality scores**
Albanesi et al., 2021 [30] Italy	Title: Evaluating interventions with victims of intimate partner violence: a community psychology approach Aim: To analyse interventions provided to victims of IPV by a Support Centre for Women (SCW) in Italy and understand its contribution to women’s empowerment using the Empowerment Process Model (EPM) by Cattaneo and Goodman	10 Women survivors of IPV who were mothers, who had turned to the Support Centre for Women (SCW) for help between 2015 and 2017, were still in contact with the Centre and willing to participate in the research (sample was self-selected as only women still in contact with the organisation were asked to participate. (Based in Italy)	Qualitative Study	Semi-structured interviews	Thematic analysis	1. The SCW provided emotional support and information to women victims. Once the immediate need for support, security and accommodation was met then the women were empowered through acquisition of skills, knowledge, and self -efficacy 2. Length of time in finding housing and accessing legal representation was challenging due to structural limitations and bureaucracy. This was a major barrier to their empowerment process	7/7 using MMAT Screening Question and Q.1 -Qualitative
Bonnycastle et al., 2021 [31] Canada	Title: Re-establishing their lives: Issues Relating to Affordable Housing for Women Escaping Violent Intimate Partner Relationships in Northern Manitoba Aim: To explore the journeys women, make as they seek safety and shelter for themselves and their children, and their reasons for making these transitions	14 (13 Indigenous) mothers who experienced abuse, needed housing, and were residing in women’s shelter at Winnipeg and Thompson. The average age of participants were twenty-eight years, and 93% came from a First Nation community	Qualitative Study	Semi-structured interviews	Cyndy Baskin’s medicine wheel (2007) as a conceptual culturally appropriate framework to collect and analyse data	1. Lack of affordable housing and lack of coordinated service response affect women’s ability to escape violent relationships. Absence of affordable housing and formal support particularly for First Nations People (mothers and their children) 2. Women make geographical moves to seek shelter and safety and complexity of this transition 3. A need for proactive and coordinated service responses to violence against women within the communities	7/7 using MMAT Screening Question and Q.1 -Qualitative
Clough et al., 2014 [32] USA	Title: “Having Housing Made Everything Else Possible”: Affordable, Safe and Stable Housing for Women Survivors of Violence Aim: To explore abused women’s experiences accessing affordable, safe, and stable housing	11 Participants were invited to complete a face-to-face in-depth interview. A sub-sample from SHARE (Safe Housing and Rent Evaluation) study	Qualitative Study	Semi-structured Interviews	Descriptive thematic analysis based in both interpretive description (Thorne, et al., 1997) and naturalistic inquiry (Aronson, 1994)	1. There is a lack of housing resources impacting on women’s ability to leave and stay safe and keep children safe 2. Survivors face multiple systemic or individual barriers to housing e.g., unscrupulous landlords, poor credit history, needing to repeat their story to each service provider re- victimizing women, a lack of tailored support 3. Survivors develop and utilize an array of creative and resourceful strategies when access to housing is limited e.g., couch surfing and working with multiple service providers to obtain funds 4. Survivors identified a variety of supportive services specifically tailored to address their needs	7/7 using MMAT Screening Question and Q.1 -Qualitative
Meyer & Stambe, 2020 [33] Australia	Title: Mothering in the Context of Violence: Indigenous and Non-Indigenous Mothers’ Experiences in Regional Settings in Australia Aim: To examine the experiences of Indigenous and non-Indigenous mothers affected by DFV in regional Queensland, Australia	17 women identifying as mothers and residing in one of the two regional Queensland locations at the time of data collection participated. A community women’s group discussion was also facilitated by two female community Elders and data recorded	Qualitative Study NOTE: The data presented in this article are based on a wider study on women’s experiences of DFV and related homelessness conducted between 2014 and 2016	Semi-structured Interviews	Thematic analysis (Braun and Clarke)	1. Domestic and Family Violence (DFV) had an immediate and long-term effects of on mothers and children 2. Experiences of financial disadvantage were worsened by the limited opportunities available in regional settings 3. Indigenous and non-Indigenous mothers both faced discrimination from realtors and landlords as a single mother of multiple children, due to having more kids’ Indigenous mothers were more disadvantaged. Indigenous women also experienced cultural disconnectedness and marginalization 4. Feeling of entrapment and post-separation abuse due to shared parenting regardless of cultural background although Indigenous mothers were more affected by overcrowding, broader experiences of family violence and undermining of her identity as a ‘good mother” page 12 5. Indigenous mothers had limited opportunities to leave without needing to give up ties to culture, country and community	7/7 using MMAT Screening Question and Q.1 -Qualitative
Sullivan et al., 2019 [34] USA	Title: Flexible funding as a promising strategy to prevent homelessness for survivors of Intimate partner violence Aim: To examine impact of funding for housing and stability and impact on survivors of DV	55 (53 were women 96% most who had children) IPV survivors who contacted the District Alliance for Safe Housing (DASH) in Washington, D.C., for housing assistance between March 2014 and August 2015, and who received flexible funding to facilitate either remaining in their homes or obtaining safe and permanent housing	Qualitative Study	Semi-structured Interviews	Using both case-oriented analysis and variable-oriented analysis (Miles et al., 2014)	1. The grant ranged from US$300 to US$8000 and of the 55 grants, 26 (47%) were for rental assistance and others grants included utility bill, security deposit and childcare and vehicle related needs 2. 50 out of 55 participants had ascertained their housing status 6 months after the funding and 3 survivors were homeless. Thus, showing flexible funding can prevent homelessness 3. the grant provided stress relief, improved ability to parent their children and get back ‘on track’	7/7 using MMAT Screening Question and Q.1 -Qualitative
Warren & McAuliffe, 2021, [35] Australia	Title: Homelessness and domestic and family violence in Queensland mining communities: The experiences of women and families accessing safe and affordable housing Aim: To provide a regional and rural frame of reference for understanding structural factors affecting women and families experience of homelessness and DFV in mining communities	People from rural & regional mining communities who had experienced homelessness as well as homelessness service providers, generic human service providers & other community representatives. Forty-three participants, 12 of whom had experienced homelessness and this included five women who had experienced homelessness, aged between 27 to late 40’s. Thirty-one participants were recruited to the research & represented 7 specialist homelessness services & 17 community services	Qualitative	In-depth interviews & focus groups	Systematic coding & thematic analysis	Recurring themes included: 1) Relocation to mining community to be with partner or family 2) Episodes of relationship & family breakdown and DFV. 3) Housing stress, risk of homelessness & homelessness 3) Exit from homelessness contingent upon access to social or affordable housing	7/7 using MMAT Screening Question and Q.1 – Qualitative
Mixed method studies							
**First author** **Year &** **Country**	**Title and Aim of the study**	**Participants in study**	**Study Design**	**Data collection**	**Data analysis**	**Findings / outcomes (if reported)**	**Quality scores**
Jonker et al., 2014 [36] Netherlands	Title: Appropriate Care for Shelter Based Abused Women: Concept Mapping with Dutch Clients and Professionals Aim: To gain insight into the perspectives held by abused women and professionals with regard to appropriate care in Dutch women’s shelters	Purposive Sampling. 56 clients and 51 professionals at the 12 participating organizations participated Clients’ eligibility criteria: (a) be 18 years or older; (b) understand Dutch, Turkish, or Arabic; and (c) have sought assistance from a women’s shelter during the past 6 months because of IPV by a partner or ex-partner Professionals’ inclusion: being chosen by the leadership and having number of years’ experience working in the shelter	Mixed Method (Qual ➔ Quan)	3 1 h brainstorming sessions (1 with clients, 1 with social workers and 1 with other staff members)	The quantitative concept mapping method (W. M. K. Trochim, 1989)	11 clusters of appropriate care within the first 6 months in a women’s shelter for abused women in order of priority: 1. Help with finding housing if necessary 2. Safety and suitable care for the children 3. Personalized, respectful approach 4. Work according to a systematic plan 5. Stopping violence in the family system 6. A transparent and safe shelter environment 7. Health and empowerment 8. Help with legal matters 9. Strengthening individuality and independence 10. Coordination of care; assistance with work and learning activities 11. Information about cash advances	7/7 using MMAT Screening Question and Q.5 – Mixed Method
Nnawulezi et al., 2018 [37] USA	Title: The influence of low-barrier and voluntary service policies on survivor empowerment in a domestic violence housing organization Aim: To examine how low-barrier and voluntary service policies influenced staff behaviour and how these behaviours then related to survivor empowerment	Purposive Sampling Phase 1 – 12 Staff members were invited to participate if they (a) had provided direct services to survivors or provided direct supervision to advocates who provided direct services; (b) had been employed for at least 2 weeks; and (c) were currently employed at the time of data collection Phase 2- 33 Shelter residents were invited to participate if they were over the age of 18 and if they had been receiving services for at least 2 weeks	Mixed Method (Qual ➔Quan)	Phase 1 – Semi-structured interviews Phase 2 – Structured Interviews	Phase 1 – Inductive Thematic Analysis (Braun & Clarke, 2006) Phase 2 – Data were analysed using SPSS Version 21	1. Phase 1 – Values and impact of low-barrier and voluntary service policies: Inclusivity and believe in the guiding values had a positive impact and easy access to services provided a sense of freedom 2. Phase 2—survivors agreed that the agency employed low-barrier policies to gain entry housing services with 91% reporting that it was true that staff trusted that they were telling the truth upon intake - Survivors reported relatively high levels of empowerment as well as safety-related empowerment 3. The hypothesis that survivors would report higher levels of empowerment when they perceived having greater autonomy with their program involvement was partially confirmed	7/7 using MMAT Screening Question and Q.5 – Mixed Method
Nnawulezi et al., 2019 [38] USA	Title: Examining the Setting Characteristics that Promote Survivor Empowerment: A Mixed Method Study Aim: To explore how organizational characteristics contributed to empowering practice, and how this practice subsequently promoted survivor empowerment	Purposive Sampling 12 Staff – Employees were eligible if they (a) provided direct services to survivors, or provided direct supervision to employees who provided direct services to survivors, (b) had been employed at DASH for at least 2 weeks, and (c) were currently employed at DASH 33 Survivors—over the age of 18 and had been living at DASH’s residential program for at least 2 weeks	Mixed Method (Qual ➔Quan)	Phase 1 – Semi-structured interviews Phase 2 – Structured Interviews	Phase 1 – Inductive Thematic Analysis (Braun & Clarke, 2006) Phase 2 – Bayesian confirmatory factor analysis (Song and Lee 2012)	1. Phase 1—DASH employee believed they were survivor-centred, mission-driven, distinctive, and assumed survivors’ right to self-determination. Setting leaders encouraged autonomy and creativity. Policies and procedures were flexible, which communicated trust, and there was high quality internal support for advocates 2. Phase 2- Survivors most highly endorsed sovereignty, compassion, and accountability practices. Survivors believed that staff were highly engaged in practices that supported their personal autonomy and trusted them and their decisions 3. Correlational analysis suggested that DASH model practices and safety-related empowerment scales were significantly and positively associated 4. Staff belief in their service’s ability to provide services were positively associated with empowerment and safety-related empowerment of the survivors 5. Empowering setting may also independently contribute to survivor empowerment	7/7 using MMAT Screening Question and Q.5 – Mixed Method
Thomas et al., 2015 [39] USA	Title: “I Have Lost Everything”: Trade-Offs of Seeking Safety from Intimate Partner Violence Aim: To explore safety-related trade-offs made by survivors of DV	Purposive Sampling 301 female survivors seeking DV services across three states located in the Northeast region of the United States. Participants were eligible if they were (a) aged 18 or over, (b) English or Spanish-speaking, and (c) receiving services from a residential or community-based DV program	Mixed Method (Qual ➔Quan)	Survey—self-administered, open-ended questions	Conventional content analysis (Hsieh & Shannon, 2005)	1. An emphasis on safety over other needs—common in a human services landscape composed of multiple narrowly defined service silos—can force survivors into a zero-sum trap of painful and detrimental trade-offs 2. Findings support the growing understanding of safety as complex, but also refine that understanding by conceptualizing safety seeking as involving extraordinarily painful trade-offs 3. Seeking safety triggered new problems for themselves and/or their families such as losses in their emotional and physical safety (30.3%), level of social support (20.6%), financial stability (19.4%), sense of home and rootedness (19.4%), ability to parent (15.8%), and freedom (12.7%)	7/7 using MMAT Screening Question and Q.5 – Mixed Method
Wood et al., 2023 [40] USA	Title: “I Felt Better When I Moved Into My Own Place”: Needs and Experiences of Intimate Partner Violence Survivors in Rapid Rehousing Aim: To expand the knowledge base related to IPV survivors in rapid rehousing, including services provision	31 survivors using vouchers facilitated by an IPV program in the U.S. Southwest	Study design: Mixed Method (Qual ➔ Quan)	Data collection: Structured Interviews	Data Analysis: Thematic analysis methods were used for qualitative interview data (Braun & Clarke,2006). Quantitative survey data were analysed via descriptive statistics, including frequencies and measures of central tendency, to assess the frequency or severity of reported barriers and experiences	1. Four major themes guiding rapid rehousing residents in IPV programs needs and experiences: the experience of getting to housing; managing resource, health, and safety needs while at rapid rehousing; accessing services to meet needs; and barriers that impeded goals 2. Virtually all participants noted personal and environmental improvements when they moved from shelter into their rapid rehousing unit 3. Over 53% of participants screened positive for moderate to severe depression, while 66% screened as having likely PTSD on validated scales used for the study 4. Many participants experienced ongoing safety concerns from the abusive partner, including stalking and threats and wanted longer term support from IPV program	7/7 using MMAT Screening Question and Q.5 – Mixed Method
Quantitative studies							
**First author** **Year & Country**	**Title and Aim of the study**	**Participants in study**	**Study Design**	**Data collection**	**Data analysis**	**Findings / outcomes (if reported)**	**Quality scores**
Sullivan et al., 2023, [41] USA	Title: Impact of the Domestic Violence Housing First Model on Survivors’ Safety and Housing Stability Aim: Hypothesis: That survivors receiving mobile advocacy & flexible financial assistance would show greater improvement at six months compared to survivors receiving services as usual. To evaluate the short-term efficacy of the DVHF model	Purposive Sampling 406 female survivors were referred who received services from five DV organisations (2 urban & 3 rural) in the United States. Participants were eligible if they were (a) experiencing recent IPV, (b) English or Spanish-speaking, and (c) receiving services from a residential or community-based DV program (d) homeless or unstably housed. Study participants were predominantly female (97%). Age ranged from 19 to 62	Quasi-experimental	Agency data and Structured interviews	Inverse-probability-weighted regression-adjustment models (Austin & Stuart, 2015) A seven-item Housing Instability Scale (HIS) was created specifically for this study. Inverse probability-weighted regression-adjustment (IPWRA) models (Austin & Stuart, 2015), comparing those who received the DVHF model with those receiving service as usual (SAU)	1. Survivors who received the DVHF model reported significant improvements in housing stability compared to those who had received SAU. Those who received services had fewer barriers & greater assets at baseline. Less likely to live with abuser, less likely to be in foster care, less likely to report barriers to housing, less likely to have stayedwith friends & family to avoid homelessness, better able to make ends meet, experienced less abuse, less likely to misuse drugs &alcohol, had higher quality of life & greater housing stability in comparison to those who received standard care. More likely to be parenting children. Less likely to experience economic abuse 2. No significant group differences were found for any other form of abuse, and survivors across both groups noted a significant decline in violence between baseline and 6 months 3. logistic regression results indicated that minority status was not a significant predictor of services received	7/7 using MMAT Screening Question and Q.3 – Quantitative – non-randomised

## Results

### Characteristics of the studies

A summary of the 12 articles that met the inclusion criteria is presented in Table 3. Six of the articles were qualitative studies, one was quantitative, and five were mixed methods studies. The MMAT quality scores are identified in the table. Although this method does not use numerical scores to determine quality, it was agreed by the authors to use seven as the maximum total score in line with the two questions asked for all studies and five questions assessed for the qualitative and mixed methods studies. The MMAT scores were compared between three authors and consensus was achieved.

### Integrative review analysis

Using the Whittemore and Knafl [28] steps we analysed 12 research articles which met the inclusion criteria. This included six qualitative, one quantitative, and five mixed methods studies. The first step of analysis involved becoming familiar with the data from the identified nine papers and this involved populating and dividing the articles’ content into separate qualitative and quantitative spreadsheets. This process involved tabulation of the studies to identify aims, participants, methods, design, data collection, analysis and findings or outcomes to provide a better understanding of the nuances of each of the articles. The second step of the analysis involved identification of initial codes that were reported by the authors of each study regarding the experiences of women leaving DV situations and seeking safe housing. The third step of the analysis involved populating relevant information from the articles under the coded headings that were compiled from the previous step. The fourth step involved reviewing themes and comparing and amalgamating the overlapping themes with all authors. This resulted in the fifth step of refining, recategorizing and naming the final overarching themes and subthemes and presenting an overall analysis. Some of the themes and sub-themes were named from the women’s words in the articles reviewed. The overarching themes and subthemes are identified in Table 4.

**Table 4:** Themes and subthemes identified in integrative review

Themes	Sub-themes
1. Experiences of leaving domestic violence	1.1 The losses
	1.2 Ongoing contact with the perpetrator
2. Barriers to achieving safe housing	2.1 Financial insecurity
	2.2 Being judged by others for leaving
	2.3 Service availability
3. Facilitators to achieving safe housing	3.1 Support, partnership, and collaboration between women and service providers
	3.2 Feeling respected and heard
4. The road to recovery	4.1 Being a good mother
	4.2 Empowerment after leaving domestic violence

Two of the 12 articles were led by Nnawulezi et al. [37, 38] which were both mixed methods studies that used an exploratory sequential design. Seven of the articles were from the United States of America [32, 34, 37–41], two from Australia [33, 35], one from Canada [31], one from the Netherlands [36] and one from Italy [30].

#### Experiences of leaving domestic violence

Experiences of leaving DV situations were addressed in nine of the included articles. Two sub-themes capture the women’s experiences. ‘*The* *losses*’ that women and their children experience when leaving DV situations are explored in the first sub-theme. This is followed by the sub-theme, *‘ongoing contact with the perpetrator’.*

##### The losses

A number of losses for women leaving DV situations were addressed across nine of the included articles. A mixed methods study exploring safety-related trade-offs from the perspectives of 309 female survivors seeking safety through DV services in the USA revealed several losses occurred [39]. The six key losses identified were “loss of emotional and physical safety for self and loved ones; loss of social support; loss of financial stability; loss of home and rootedness; loss of control over parenting; and loss of freedom” [39, 39]. Two of the studies [35, 40] highlighted the loss of access to health services that women experienced due to conditions such as diabetes and mental health conditions. This loss came at the expense of their own health as they did not have time or could not afford medication and was coupled with a lack of mental health resources [35, 40].

Seven of the articles highlighted the loss of home, community and rootedness and not being able to return to their own community, especially when the women came from isolated rural and regional areas. DV often resulted in women and children having to leave their family home, seeking refuge in women’s shelters [31, 35, 40] or residence in poor quality housing [35, 40], where they continued to feel unsafe [32, 40, 41]. For some women and children, leaving the DV situation resulted in homelessness due to a lack of affordable housing options [31, 32, 34, 35, 39, 41]. Alternatively, to attain safe, affordable housing, some DV survivors were forced to relocate, [35, 40], experiencing a loss of belonging to a community [31, 39].

Four articles identified that women often make geographical moves to seek safety and shelter and the complexities of this transition. Wood et al. [40] describe women relocating to a different state so they could be away from their abusive partner which subsequently meant being away from supportive networks and living in violent communities. Bonnycastle et al. [31] discussed the geographical remoteness of moving away from their First Nations community for safety. Similarly, Meyer and Stambe [33] report that moving into independent housing post-crisis accommodation proved difficult for women in regional settings. Cultural background further complicated women’s experiences [33]. Indigenous mothers in Meyer and Stambe’s [33] study further discussed experiences of being forced to consider substandard housing in the absence of available public housing and an inability to compete in a limited, regional housing market.

Thomas et al. [39] found that for women seeking safety it also meant relocating their home and community which led to an actual loss or a sense of loss in rootedness. This resulted in difficulties their children would face if “uprooted,” especially regarding friends and school. Overall, the use of phrases such as “having to start over” and “I have lost everything” suggest that the loss of home, relocating to another community and uprooting their children equals a complete overhaul of one’s life to get away from their abusive partner [39]. Women came to the realisation that they had to move with uncertainty about the future due to the fear of their children being hurt or abused [39].

Women in Bonnycastle et al.’s [31] study identified the importance of having your own space at home and that culture and language provide a sense of identity at home. Housing unavailability often led to overcrowded living conditions [31, 40]. Similarly, Albanesi et al. [30] also found that co-housing with other women was difficult due to cultural and structural reasons such as a lack of private space and forced intimacy.

##### Ongoing contact with the perpetrator

Five of the articles identified ongoing contact with their perpetrator after leaving a DV situation being a major source of concern. Re-traumatisation, disrupted healing and ongoing manipulation by the perpetrator were experienced by many women and their children [33, 35, 39]. Six participants in a mixed methods study reported heightened fear and stress when required to communicate with their perpetrator for their children’s needs, and during exchange of children’s care where shared custody arrangements were in place [39]. Twenty-six participants in the same study experienced a loss of control over parenting capacity, as well as fear and worry for their children’s safety, where abusive partners sought and obtained partial or full custody of their children. One woman feared for her life, this fear continuing after she left the relationship but had to remain in close vicinity to the perpetrator [35]. Similarly, Albanesi et al. [30] found that women reported fear about being chased by the partner, because even if the partner was unaware about where they resided, they knew where their children went to school and where the woman worked.

Ongoing contact with their perpetrator was also an issue for women survivors who lived in a small and/or rural community or had no informal support beyond their violent partners family. Seeking safe housing in a women’s shelter within their community meant they remained near their perpetrator and his family, creating an inability to feel free of the fear of their partner. For some participants, their only option of secure housing was with their partners’ family, intensifying tensions with their violent partner and with other family members [31]. The experiences of women survivors in leaving DV situations are complex. Across both sub-themes in this section, securing safe living arrangements was of paramount importance to the successful recovery of women and children leaving DV situations.

#### Barriers to achieving safe housing

Eleven of the articles discussed the barriers to achieving safe housing when considering women’s experiences of escaping DV.

##### Financial insecurity

Eight of the 11 articles discussed women’s experiences with financial insecurity when leaving DV situations. In Clough et al.’s [32] study, stable, affordable housing was critical in increasing safety for women and their children and impacted their ability to leave and stay safe. Women needed financial assistance to find safe housing and this resonated with other studies’ findings [30–32, 36]. Survivors faced multiple systemic or individual barriers to housing including unscrupulous landlords and poor credit history [32].

Financial insecurity was also an issue for women not having a job and their role in looking after children [30, 33, 34, 39, 40]. Thomas et al. [39] identified women’s loss of financial security due to the loss of the abusive partner’s income and the added cost of relocation and entering into a shelter. Wood et al. [40] identified that having the means to pay for permanent housing and time pressures was a constant anxiety. Two of the studies conducted by Meyer and Stambe [33] and Bonnycastle et al. [31] identified that experiences of financial disadvantage were worsened by the limited opportunities available in regional settings and the geographical remoteness of some areas. These same two studies [31, 33] highlighted the absence of affordable housing particularly for First Nations People being more disadvantaged. Both Indigenous and non-Indigenous single mothers faced discrimination from realtors and landlords due to having multiple children [33]. One Australian study highlighted the disadvantages of women on low incomes escaping DV as being unemployed meant that they have no chance of gaining a place in a share house [35].

##### Being judged by others for leaving

Three of the articles identified the loss of respect felt by women when leaving DV situations. Albanesi et al. [30] found that women often felt judged by other support services such as social workers and police. Similarly, Nnawulezi et al. [38] showed that staff in their study agreed that many other formal helping systems for women experiencing DV disrespected, policed, and discriminated against survivors. Participants from two research studies shared their feelings of being re-victimised or feeling judged and blamed by services that were meant to support them [30, 32]. Qualitative data from a mixed methods study in the USA alluded to similar barriers, often created by services with obstructive screening policies [38].

##### Service availability

Four of the articles identified the lack of service availability that contributed to their vulnerability. Bonnycastle et al. [31] also identified that women felt unsupported by formal supports (notably First Nation or chief and members of council, law enforcement, and the child welfare system). This could possibly be explained by service providers working in housing, social service or DV agencies being under-resourced, uninformed or unable to respond effectively to the safety and housing needs of survivors. Subsequently, this results in women having to visit multiple offices and with each visit being required to repeat and validate their history of DV [32]. This often results in women finding it difficult to establish trust with services [30, 39].

Bonnycastle et al. [31], report that informal support from family and friends was not always a viable option, and that seeking formal support was fraught with difficulty. In the same study, some participants revealed that there were little to no formal DV services within their home communities, and where DV services were available, they were often understaffed. A further barrier relevant to feeling judged was that accessing formal support services was only available after an episode of violence, and was governed by restrictive policies based on cultural values and beliefs, nepotism [31], and service bureaucracy [30, 32].

#### Facilitators to achieving safe housing

Ten of the articles discussed the facilitators to achieving safe housing when considering women’s experiences of escaping DV.

##### Support, partnership and collaboration between women and service providers

Formal support, including safe housing, resources, psychological support and informal support that included family and friends,’ were an important road to recovery for women when escaping DV and achieving safe, sustainable housing. Albanesi et al. [30] identified formal supports as essential, to ensure housing solutions that led to safe housing and protection from the perpetrator. This formal support also included information about resources which led to increased access to legal support and services. Women were then ready to increase their skills which included self-actualisation [30]. Participants in Wood et al.’s [40] study participants also overcame housing barriers by paying back debt, and accessing legal help. For participants in Sullivan et al.’s [41] study, survivors who received support from the DV Housing model, reported significant improvements in housing stability in comparison to those receiving standard care. Similarly, Clough et al. [32], Bonnycastle et al. [31], and Jonker et al. [36] identified that stable, affordable housing was critical in increasing safety for the survivor and her children, and women needed financial assistance to find safe housing. Four of the studies also identified that professionals ought to help with financial matters as well as legal procedures [30–32, 36].

The importance of informal support was highlighted in three of the studies [30, 31, 39] as provided by family, friends and colleagues that could assist with practical and financial issues such as loans and physical, emotional, and social support from family and friends. The normalisation of these supportive relationships provided the opportunity for intimacy and positive experiences [30]. When women left their abusive partner, informal support systems were affected by their safety-seeking efforts resulting in women losing their support systems [30, 39].

Numerous papers reported findings of support from service providers as an essential facilitator to accessing safe housing for women and children leaving IPV situations. Support in linking women to other supportive agencies, finding suitable accommodation and coordination of care and assistance with work, and learning activities were considered important facilitators [30, 31, 36]. Professional support for assisting with establishing child care arrangements was also reported as beneficial [36]. Such formalised support and services were reported as best provided as a multi-pronged, collaborative approach [36]. Women who received support from housing agencies also reported experiencing less violence and economic abuse than those receiving standard care [41].

Women felt that a safe home “was more than just four walls and a roof “. Home was identified as a connection to family, community, culture, and safety. Culture and language were viewed as providing a sense of identity and belonging. Being able to secure a safe home within their community served to provide the women and children with their own space as well as rootedness. This key finding is emphasised by another study which built on two previous studies by the same authors. The earlier studies first interviewed women about their practical and emotional support needs during their stay in a women’s refuge, and then again six months later in their new lives in independent housing. The most recent study shared findings of re-interviewing 12 women five to seven years later, who were participants in at least one of the previous studies. The participants revealed that when at home, women identified the importance of having their own space at home [31].

Seven of the studies in this integrative review [30–32, 34, 36–38] highlighted the importance of partnership and collaboration between women and service providers in addressing DV towards women. As important to establishing supportive partnerships between women survivors and service providers were low-barrier and voluntary service policies. Three studies identified organisations that had low-barrier and voluntary service policies. Such policies resulted in a smoother transition for DV survivors into affordable and safe housing [34, 37, 38]. Low-barrier policies are defined as a “compilation of specific policies designed to reduce the eligibility requirements that can be barriers to accessing services” [37, 37, 38].

Trust was also noted as essential as a facilitator of partnership and collaboration between the DV survivor and service provider. Five of the included papers highlighted that trust between the woman and the service provider was essential in facilitating safe housing and a successful, secure future. Trust was reported as established through procedural flexibility in decision making about services, and the supports and needs of the woman and her children [31, 32, 34, 36, 38]. Further, the mutual establishment of goals, with a ‘one step at a time’ approach, was reported as essential to the facilitation of women’s trust in the formal services [30, 36].

Trust between the women and children and IPV supporting services was a two-way process. All participants in Nnawulezi et al.’s [37] study noted that it was as equally important for the service provider to trust the women survivors as it was for the women to trust the service provider. The success in the provision of implementing low-barrier and voluntary service policies mutually trusting relationships was an integral part to implementing these core activities between the women survivors and the service provider [38].

##### Feeling respected and heard

Five of the studies in the integrative review [30, 32, 34, 36, 37] identified the importance of feeling respected and heard in their journey to recovery from leaving a DV situation. Feeling respected and heard by other DV survivors as well as service providers were important facilitators in accessing DV services and securing safe housing. Women who were able to build positive relationships with other women who had similar experiences reported feeling respected and heard. These relationships improved psychological wellbeing and resulted in increased self-efficacy and the forming of positive relationships [30]. Two studies reported that these factors were instrumental in achieving stability, including safe housing [30, 32]. Participants in Clough et al.’s [32] study describe feeling respected and validated by well-trained, compassionate DV workers. Positive experiences with DV services were noted as non-judgemental emotional support; protection and safe shelter; development of the women’s awareness of the violence as not their fault; and building of the women’s self-esteem, self-awareness, empowerment and overall well-being [30].

The importance of engaging in empathetic and nonjudgmental listening, highly relevant to feeling respected and being heard was highlighted in four of the studies. Listening deeply to survivors’ needs was an imperative part of practice when implementing policies. Participating service providers in these studies highlighted that listening to, and hearing, women survivors’ reported needs ensured that organisational programming aligned to what survivors wanted throughout safe housing service provision [32, 34, 36, 37].

#### Road to recovery

Nine of the articles examined the road to recovery when contemplating women’s experiences of escaping DV to attain safe housing. Within this theme, ‘being a good mother’ and ‘empowerment after leaving DV’ were deemed as essential to the recovery of the women and her children, and closely linked to securing safe housing.

##### Being a good mother

The importance, pressure, and responsibility experienced by DV survivors to be a ‘*good mother’* and able to parent their children with safety on their road to recovery was a sub-theme across seven articles [30–34, 36, 39]. Being a mother added an additional layer of complexity as their needs to improve their currently unsafe situation, increase their skills to secure economic independence, develop self-esteem and improve overall psychological well-being were inextricably linked to providing safety for their children, and seeing themselves as ‘good mothers’ [30, 31, 33, 34]. Participating mothers in a mixed methods study reported the challenges of juggling finances, time, and ability to care for their children while seeking safety from their perpetrator [39]. An Australian study reported on the experiences of nine Indigenous and eight non-Indigenous mothers. Their experiences included feeling the responsibility of ensuring the safety and wellbeing of their children. For Indigenous participants, their identity as a ‘good mother’ was further challenged by social constructs of overcrowded housing, higher rates of family violence, and greater child protection interventions in comparison to their non-Indigenous counterparts [33].

Three articles discuss the importance of being able to protect, and mother children after leaving DV situations. Safety and suitable childcare for children was found to be the second highest priority in Jonker et al.’s [36] study which identified 11 priorities for women leaving DV situations. Sullivan et al.’s [34] study, found that grants including rental assistance and payment for bills increased women’s ability to parent their children and get back on track. Clough et al.’s [32] study identified that women used whatever was available to ensure a safe environment for their children whilst looking for stable housing. Women used and developed creative strategies to manage complex situations to reduce levels of trauma and stress for their children, such as couch surfing and working with multiple service providers to obtain funds [32].

##### Empowerment after leaving DV

Four research studies [30, 36–38] identified the impact and importance of empowerment for women after leaving DV situations and finding housing. Nnawulezi et al. [37, 38] showed that survivors who had greater autonomy in a shelter program demonstrated higher levels of empowerment. Two other studies concurred, reporting that after immediate needs for support, security and accommodation were met, women were empowered through skills and knowledge acquisition and self-efficacy [30, 36]. Jonker et al.’s [36] study showed empowerment was the seventh highest need for women after leaving a violent relationship and finding safe housing.

## Discussion

The integrative review aimed to explore women’s experiences of escaping DV and achieving safe housing. There were key facilitators for DV survivors in leaving DV situations and securing safe housing. This discussion will focus on the key barrier of *‘The consequences of leaving DV situations’* as well as key facilitators, captured as ‘Being connected to support mechanisms’, and ‘Empowering women regaining their lives with their children’. All of these factors can influence the woman’s decision, and capacity, to leave the violent relationship and secure safe housing.

### The consequences of leaving DV situations

Key consequences identified by this review were the increased vulnerability of women with children, the long-term effects of the ongoing contact with the ex-partners, and financial insecurity. Two-thirds of the articles in this review revealed that women experience many losses because of leaving DV relationships and this may include emotional, physical, financial constraints and loss of control over continuing relationships with perpetrators that involve their children [30–35, 39, 40]. Women have been shown to experience a heavy sense of loss when subjected to DV and unable to control emotions. Women have experienced psychological problems caused by the long-term DV from their partners [42]. Similarly, Māori women in Wilson et al.’s [43] study reported a loss of control over their continuing relationships with their partners and their children as a barrier to leaving a violent relationship. They recognised the control exerted by their partners exacerbated threats to the women’s life and safety and took a toll on the women’s psychological and emotional wellbeing, diminishing their sense of self-confidence [43]. Another study [44] in Iran, has identified that women who have been subjected to violence by their husbands faced challenges that related to their psychological health. Women have also been afraid of the perpetrator’s reaction if they find out about her disclosure about DV to health care practitioners [45].

Challenges have been identified in finding accommodation for women experiencing DV due to staff shortages and the availability of appropriate resources and DV services. These situations are often exacerbated by isolation, long distances, and lack of transport for women experiencing DV [46]. As identified in this paper, some included studies linked self-confidence and autonomy to women IPV survivor’s success in securing safety and stability, including safe housing, for themselves and their children [30, 36–38].

This integrative review also highlighted the loss of belonging, and rootedness that First Nation peoples experienced due to leaving their tight-knit communities [31, 33]. Similarly, Māori women in Wilson et al.’s [43] study who decided to leave were faced with challenges leaving their homes, due to the isolation from friends and families. This resulted in women experiencing vulnerability when unsuccessful in asking for help from friends, family or agencies [43]. The importance of culturally safe, responsive and trauma-informed care has been highlighted to ensure that the needs of First Nations people experiencing DV are met [47].

Women leaving DV situations often experience continuing contact with the perpetrator due to their children’s ongoing custody arrangements and concern for their children’s safety when in the care of their abuser [30, 31, 33, 35, 39]. Supporting this as a key barrier to leaving IPV relationships for safer living options, participants in a Canadian qualitative study revealed apprehensions about facing legal custody processes, and fear of shared custody where they had witnessed the perpetration of violence towards their children [48]. Studies that have explored the use of the legal system, including child custody processes by abused women who have children have reported that children can prevent women from pursuing legal prosecution of their perpetrator, due to concerns about their children’s safety and wellbeing [49, 50]. Further research is needed on how such barriers can be navigated and women who are mothers supported in providing safety for themselves and their children where the IPV perpetrator is allowed parental custody.

Financial insecurity can result from women experiencing DV situations [30–34, 36, 39, 40]. Housing instability and exposure to DV also compromises women’s sexual and reproductive health by restricting contraceptive access that may result in unintended pregnancy [51]. Grace et al. [51] found in their study that the majority of participants did not use contraception, however, this may have been due to financial instability as one in five women was unable to afford health care and all experienced housing instability as a result of leaving a DV situation.

One study [52] found that the longer the woman remained in the relationship, the finances were more tied up between the partners. Another study [53] found that women were financially dependent and did not earn their own income. Despite the abuse, some women were thankful for their partners’ support throughout the years [53]. Therefore, making the public and health professionals aware of legal advice and financial support that is available from domestic violence services is crucial in overcoming this barrier [52]. Learning income-generating skills is important to reduce economic dependence of the woman on her partner and increases maternal financial independence [54].

This integrative review also identified women feeling unsupported by formal supports and being judged by others for leaving the DV situation [30–32, 38, 39]. Women have also feared about being judged for not leaving a DV relationship, and not wanting to be stigmatized from others including health care practitioners [45]. Similarly, a systematic review [55] found that victims experiencing DV feared being judged by their friends, family, neighbours and health care providers as a barrier to disclosing that they were in that situation. Carthy and Taylor [52] also found the social stigma of not wanting to disclose DV created an additional barrier to seeking help. This was exacerbated by societal pressures, and that others would think she should have known better than to put up with the abuse [52].

The impact of the COVID-19 pandemic has worsened the situation for some women with organisations having to implement social distancing and reducing the number of women able to access shelters [56, 57]. This occurred in tandem with an increase in the number of women experiencing DV during the COVID-19 pandemic due to lockdown conditions [58]. Therefore, lockdown and social distancing requirements of COVID-19 led to greater difficulty for women accessing DV services, including safe housing options [57, 59, 60]. While some DV agencies had to suspend their services altogether, other DV organisations were able to access additional government support for homelessness and housing services [61]. However, the challenge for women being able to access such services was hampered by lockdown creating an environment where many DV victims were exposed to 24-h surveillance by their perpetrators. This is further heightened by this paper’s [61] findings that a lack of support of DV services felt by women attempting to leave violent relationships existed pre-Covid pandemic restrictions, and continues post pandemic restrictions. Further, the United Nations (UN) Women Australia [62] identified that the COVID-19 pandemic not only resulted in increased levels of DV, but also substantial losses in employment and reductions in unpaid care work for women across the globe. This resonates with the identified barrier in this review of financial instability preventing women from leaving violent relationships and secure safe housing options.

There have been many lessons learnt during the COVID-19 pandemic, including those for better planning in all countries for crisis events. For DV and ensuring women’s and children’s safety, some suggestions have been to ensure resilience in infrastructure and supportive IPV services to survive and thrive during crisis, embracing digital technologies, and increasing capabilities to gather real time data and conduct rapid assessments on gender impacts in crisis situations [62]. One study [63] identified insights into ways in which practitioners pivoted services during COVID-19, to respond remotely to women experiencing DV and the challenges of undertaking safe planning and risk assessment when working on video, and phone-based delivery. These align with the key facilitators identified in this review as being connected to support mechanisms and women regaining their lives with their children after leaving an DV situation.

### Being connected to support mechanisms

Formal and informal supports were extremely important findings in this integrative review to facilitate women’s experiences of leaving DV relationships to achieve safe housing [30–32, 36, 37, 39–41]. Informal supports such as family and friends need to know what formal services are available and DV organizations should distribute information about hours of operation and who to contact so that referrals can be completed in a timely manner [64]. The importance of service providers being able to provide ongoing training about DV to workforce members and education to all people about how to respond and recognize DV cannot be emphasised enough [64, 65]. Health care systems could empower women by improving the capacity of health care providers in providing information to women about DV, especially legal issues, and supportive referral centres [54]. A recent Cochrane review [66] found that healthcare providers are ready to respond to learn about training about intimate partner violence towards women. One study [67] in India has indicated that healthcare providers demonstrated a significant increase in knowledge, preparedness and attitudes following training in responding to women’s needs escaping DV, as well as supportive practices including talking to women and validating their needs. This level of training also included integration of system-level changes that involved clinicians to deliver the training who had managerial responsibilities that ensured mentorship [67]. Women experiencing DV often need practical support such as social security benefit, housing, parenting support and finding employment and women value advocacy support as helpful in finding a house or a job [68].

In light of the previous reference about rural challenges in organizing accommodation for women due to lack of appropriate and services [46], future training could be targeted to rural areas to provide opportunities to co-train with local services that could strengthen integration, collaboration and mutual understanding. Specifically, maternal child and family health nurses are best placed to deliver care for women experiencing DV. However, greater support is required for sustainable nurse DV work, especially rural nurses who experience greater practice barriers [65]. Despite these barriers, relationship building is sometimes easier in regional and rural areas that already have existing connections with communities [46]. Similarly, Māori women in Wilson et al.’s [43] study found the support and strength of others enabled them to tolerate difficulties in leaving their violent relationships.

One systematic review [69] has shown how DV survivors benefited from support from external agencies including employment opportunities, legal aid and tangible resources such as clothing vouchers. One other helpful resource included educational information about DV and abusive relationships [69]. Enhanced collaboration between services may ensure that a culturally responsive approach may strengthen partnerships and rely less on individuals’ work practices to enhance women and childrens’ safety and wellbeing [70]. Practitioners have identified the importance of collaborating with internal team members of their organisations as well as specialist professionals external to their team, as these collaborations provide support, comfort, and specialist knowledge about social sector services and abuse. Health practitioners have highlighted how other team members have provided emotional support and inspiration to address DV [71].

In Canada, service providers and program staff have previously noted the importance of partnerships between their own service and other aspects of the system in easing referral processes. This resulted in pregnant women experiencing substance abuse being more likely to access the correct services and experience reduced service fragmentation. Sharing of program information within this system enabled information to be shared and service providers to become familiar with each other’s roles and develop trusting relationships [72]. Another review has highlighted the importance of working locally with service providers to ensure programs are contextually aligned and interventions are appropriate [73].

In Australia, for women experiencing DV in Aboriginal families, community partnerships amongst service providers have been identified to enable cross-agency work in a culturally safe environment, helping access to housing and programs for health and wellbeing. Referral pathways to other trusted service and community providers alleviates the shame for Aboriginal women experiencing DV [47]. Similarly, half of the women experiencing DV in Prosman et al.’s [68] study reported the importance of expressing themselves in their culture and language helped them to address barriers to source support more easily. Culturally sensitive support enabled them to accept help and share their sorrows more easily. Speaking in their own foreign language enhanced the bonding between the mentor mothers and the abused women [68].

One systematic review has highlighted the need to create a supportive environment for pregnant women experiencing DV [74]. Another qualitative metasynthesis [71] has shown that clinicians see their role as the most appropriate for responding to women experiencing DV as they are able to develop trusting relationships and talk to women over a period of time. They recognised continuity of care as an important component of forming strong relationships with women and being able to respond to DV [71].

### Empowering women regaining their lives with their children

One study [75] showed that women escaping DV enabled them to refocus on the child’s needs. Even though mothers and children may have endured undermining of DV over many years, positive perceptions have been demonstrated and this is testimony to resilience of these relationships. Health care providers should build on these relationships when working with women and children and create spaces to work together [75]. Empowering couples by improving couple’s life skills, and economic empowerment could reduce DV, especially during pregnancy [54].

Māori women in Wilson et al.’s [43] study found strength in their own values and beliefs. Staying strong for these Māori women experiencing DV provided a platform for reviving and healing their well-being [43]. In Sapkota et al.’s [74] review, the main component of interventions included mentoring and supportive counselling that aimed to empower women in their flight from DV. Interventions were targeted around empowerment and assisting women to disclose their experiences of abuse as well as identifying the best resources to find a solution that was most suitable with her situation. Interventions should seek to provide services that are tailored to meet the woman’s individual circumstances and needs [74].

### Limitations

We appreciate that the included studies show a diverse range of contexts of DV and IPV globally and that all of the countries represented in this integrative review may view this topic differently. Some of the studies included participants from Indigenous backgrounds (Australia and Canada) and regional areas (Australia). These studies may not be representative or generalizable to other areas of these countries.

## Conclusions

This review has highlighted the need for service and health care providers to work together and collaborate effectively with the woman experiencing and escaping DV. This means being able to receive training and education to provide her access to the most suitable educational resources and services that are most suitable for her situation. Providing women support, encouragement and counselling who are experiencing DV will facilitate their path towards recovery to achieve safe housing.

## Data Availability

The datasets generated and/or analysed during the current study are not publicly available due to this being an integrative review and data were not collected. The literature reviewed is displayed in a table within the manuscript.
